# Institutional Notes and News

**Published:** 1911-05-20

**Authors:** 


### Institutional Notes and News.

**The German Hospital Dinner.**

On Friday, the 12th inst., a large number of friends and supporters of the German Hospital, Dalston, met at the annual festival dinner. An unusually good musical programme preceded the business of the evening, which was the setting forth by Sir Frank Lascelles, the Chairman, of the needs of the institution. At present the imperative need is the enlargement and improvement of the out-patient department. Sir Frank, who takes a keen personal interest in the work of the institution, referred to the new nurses' home which is shortly to be opened, and to the various other improvements which are under consideration. The new president of the hospital is H.R.H. the Duke of Connaught, whose acceptance of the office of president once more closely identifies the Royal family with the work of this East-end hospital of which his Majesty the King is patron. This year's dinner produced a record subscription list, over £5,000 being handed to the treasurer by the various stewards. H.I.M. the German Empress intimated her desire to visit and inspect the hospital on Thursday last, and was shown over the buildings by the chief officials. Her Majesty has never lost an opportunity of showing her active interest in the work of the institution, and this is by no means the first visit she has paid to the wards.

**The Closing of a Cottage Hospital.**

The North Wimbledon Cottage Hospital, we understand, will be closed according to the present decision of the executive committee, and the building of a new hospital will be begun at once. This, it will be remembered, is an alteration of the original proposals by which the new institution was to be built in three portions. In the meantime the work of the hospital will be carried on in temporary quarters. A sum of £9,500 will probably be spent upon the new buildings, and it is generally believed that the decision of the committee to start rebuilding at once has been the result of the satisfactory sum already subscribed for this purpose. More than two-thirds of this amount is already in hand. The recent appeal in connection with the King Edward Memorial Fund realised £2,000. We are informed that the finding of temporary quarters has presented some difficulty to the committee which is grappling with the matter.

**An Appeal to the Suburbs.**

The annual meeting last week of the Central London Throat and Ear Hospital, Gray's Inn Road, was presided over by Colonel Sir John Young, C.V.O. Of the 11,404 new out-patients and 726 patients in the wards, a very large number was sent by medical men—58 per cent. of the in-patients, and 37 per cent. of the out-patients were so recommended. These figures, it was urged, indicate not only the value of the hospital but disarms any criticism as to abuse of the charity. Fifty per cent. of the patients were sent from the suburbs and provinces, but it is felt with regret and even anxiety that very little support is accorded to the hospital from the districts from whence patients came. The hospital is cramped for room in every way, the number of beds is insufficient, the out-patient department is too small. The chairman made an earnest appeal, especially to the public outside London, for assistance whereby an enlargement may be undertaken for which plans have already been prepared. The president of the hospital, her Royal Highness Princess Louise, who opened the first part of an extension scheme in 1906, sent a gracious letter indicating her continued interest in the welfare of the hospital and commended its work.

**Ground Alterations at a Buxton Hospital.**

Certain improvements are now in preparation at Buxton by which it is hoped to build new hot baths in the gardens of the Devonshire Hospital and Buxton Bath Charity. The present baths have been enlarged already, but that was in days when there was one-third only of the present numbers attending the hospital. The medical staff is of opinion that a covered way should lead to the proposed new hot baths, so that there should not be the slightest possible risk from exposure on wet or cold days after bathing. In the buildings of the hospital also a new heating system will probably be installed, towards the cost of which the late Dr. Lorimer's residuary estate, which amounts to over £5,500, will be reserved. The hospital authorities have long pointed out that the water treatment is as potent in winter as in summer, and poor patients are reminded that there is a Samaritan fund connected with the hospital which helps those in need to return home by paying the whole or a proportion of their return fare. There is an important system also whereby patients report on their progress six weeks after their discharge. This is carried out by means of postcards which are given to patients as they leave the hospital.

**Nightingale Memorial at Derby.**

The Royal Infirmary at Derby has received the honour of being chosen as the site for the statue of Miss Nightingale, which is to be the county memorial to her. The sub-committee last week, which decided this point, is now issuing an appeal for funds. The cost of the statue is expected to be £1,300, and, we understand, the co-operation of the Mayor of Derby, Sir Thomas Roe, M.P., has been gained. Another supporter of the scheme is Sir Arthur Heywood, very well known to the town and hospital of Derby, and so it is hoped that the sum will be subscribed very quickly. The decision that the grounds of the Royal Infirmary at Derby had been chosen for the site of the statue had hardly been announced when Londoners were informed that in all probability the site for their statue to Miss Nightingale had been chosen also. This, it is expected, will be opposite to the Crimean trophy at the foot of Waterloo Place.

**The New Hospital at Thirsk.**

Mr. F. E. Walker, chairman of the Rural District Council, has opened the new Isolation Hospital at Thirsk, Yorkshire. It consists of a pavilion, with two wards of four beds apiece, and a nurses' room, that, we understand, accommodates two nurses and the caretaker. Mr. F. E. Walker in opening the hospital said two or three words about its history. Like most local authorities Thirsk had been pressed by the Local Government Board to provide means of isolation in the district, and eventually after advertising for a site and getting no reply, the field in which they were met became available and in 1908 it was purchased. Since that time, after settling plans the work had gone on practically without alteration up to the present time, except for the making of a road. There had been many difficulties. The ratepayers had wanted it done at as little cost as possible, the Local Government Board wanted it done expensively, and, as it were, they had been between the devil and the deep sea. Mr. T. Stokes, of Thirsk, is the architect.

